# The Prognostic Role of the Platelet-Lymphocytes Ratio in Gastric Cancer: A Meta-Analysis

**DOI:** 10.1371/journal.pone.0163719

**Published:** 2016-09-29

**Authors:** Zhengshui Xu, Wei Xu, Hua Cheng, Wei Shen, Jiaqi Ying, Fei Cheng, Wenji Xu

**Affiliations:** Department of General Surgery, The Second Affiliated Hospital of Nanchang University, Nanchang, Jiangxi, China; University of North Carolina at Chapel Hill School of Medicine, UNITED STATES

## Abstract

**Background:**

Systemic inflammatory parameters, such as the elevator PLR (platelet-lymphocyte ratio), the NLR (neutrophil-lymphocyte ratio) and the platelet count (PLT), have been found to be associated with the prognosis in gastric cancer; however, these results, especially those relating to the PLR, remain inconsistent. So we aimed to evaluate the prognostic role of the PLR in gastric cancer by conducting and presenting the findings of this meta-analysis.

**Methods:**

We conducted a systematic literature search in PubMed, Embase and the Cochrane Library to evaluate the prognostic value of the PLR in gastric cancer. The quality of the included studies was evaluated using the Newcastle Ottawa Quality Assessment Scale (NOS). The hazard ratio (HR) /Odds Ratio (OR) and its 95% confidence were pooled using a random effects model. A funnel plot based on overall survival was used to evaluate the publication bias.

**Results:**

It total, 8 studies comprising 4513 patients with gastric cancer met the pre-setting inclusion criteria. In comparison to the normal PLR, an elevated PLR was correlated with a higher risk of lymph node metastasis with an OR of 1.50 (95% Cl:1.24–1.82; I^2^ = 17%) and serosal invasion (T3 +T4) risk with an OR of 2.01 (95% Cl: 1.49–2.73; I^2^ = 55%), and an elevated PLR also increased the advanced stage (III +IV) risk with an OR of 1.99 (95% Cl: 1.60–2.46; I^2^ = 28%). An elevated PLR was not a reliable predictor for OS with an HR of 0.99 (95% CI: 0.9–1.1; I^2^ = 12%).

**Conclusions:**

An elevated PLR was correlated with a higher risk of lymph node metastasis, serosal invasion and advanced stage (III +IV) risk in gastric cancer; however, the PLR may not act as a negative predictor for the overall survival of gastric cancer.

## Introduction

For many years, gastric cancer (GC) has been one of the most common cancer in the world and one of the leading causes of death worldwide.[1] Some methods, such as computed tomography, magnetic resonance imaging and endoscopy ultrasonography, can predict the preoperative tumor stage to some extent, however, it is not precise. Furthermore, these examinations are prohibitively expensive. In the future, we expect that an ideal marker will be developed to predict the prognosis of gastric cancer. In fact, there are no ideal predictors that can be reliably used in the clinic. Therefore, we aim to discover a reliable predictive marker, and our clinicians and researchers have been making efforts to identify this type of bio-marker. Recently, we may have had some success, as an increasing number of studies have shown that a systemic inflammatory response has a relationship with the development and progression of cancer [2–4]. Inflammation-based variables, such as the PLT count, the NLR, the PLR, etc., may be predictive markers for the prognosis of gastric cancers [5–10]. Among these markers, the PLR may be the most controversial [11,12]. Recently many studies have been performed to assess its prognostic value in gastric cancer [5,6,11,13–16]. According to their results, the prognostic role of the PLR remains inconsistent. So we conducted this meta-analysis to evaluate the prognostic role of the PLR in gastric cancer

## Materials and Methods

### Literature search

**Fig 1:**
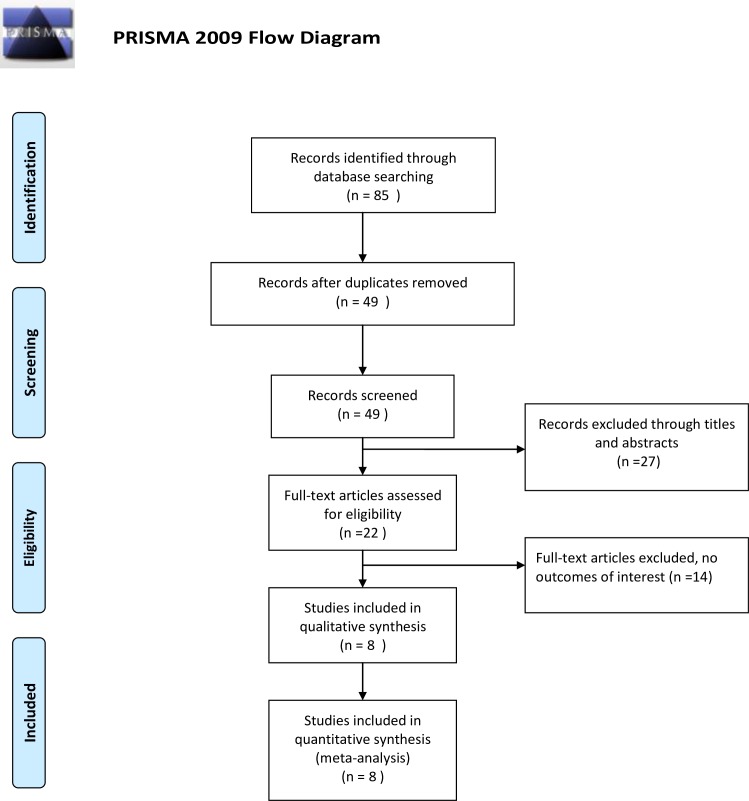
Literature screening flow chart and results. doi:10.1371/journal.pmed1000097.

We conducted a systematic literature search in PubMed, Embase and the Cochrane Library. The search for relevant studies was performed using the following terms: (“platelet-lymphocyte ratio” or “platelet-to-lymphocyte ratio” or “platelet lymphocyte ratio” or PLR) and (“gastric cancer” or “gastric adenocarcinoma” or “gastric carcinoma” or “stomach tumor” or “stomach neoplasms”). A MeSH/Emtree search for “stomach neoplasms” was also performed. (Appendix 1) Three databases were searched from inception to July 20, 2016. We scanned the reference lists of all studies that had been identified in order to search for other potentially eligible studies. All articles were assessed independently by two authors according to the eligibility criteria that we had designed. Articles were independently categorized based on their title and abstract. When articles could not be categorized based on this information, the full-text was retrieved and reviewed. Any disagreements or questions were resolved by consulting another author. According to the Preferred Reporting Items for Systematic Reviews and Meta-Analyses (PRISMA) statement [17], the selection process of the articles is shown in Fig 1.

### Inclusion and exclusion criteria

Studies were included if they met the following criteria: (a) all patients must have been diagnosed with GC by a pathological examination; (b) there was available data to be investigated (for example the number of patients and the presence or absence of lymph node metastasis, the depths of tumor invasion or stage of gastric cancer, or HR and 95%CI on the OS which was provided by the original article or could be calculated from Kaplan-Meier cumulative survival curves and the number of patients[18]); and (c) the patients had blood samples all obtained before treatment; (d) any study designs and without restriction in sample size. Studies were excluded based on the following criteria: (a) those consisting of letters, conference abstracts, review articles, and posters (b) those lacking required outcomes data that could not be calculated from the article data; (c) studies referring to any treatment (including operative or/and chemoradiotherapy) prior to their blood draw; and (d) those lacking human research.

### Data collection and assessment of methodological quality

Two investigators independently evaluated and extracted the data. Any disagreements or questions were resolved by consulting another co-author. The following information was recorded from each study: first author, year of publication, country, study design, the type of gastric cancer, age, the cut-off value of PLR, the number of patients with or without lymph node metastasis, the depths of tumor invasion or different stage of gastric cancer, or HR and 95% CI on the OS that was provided by the original articles or could be calculated from Kaplan-Meier cumulative survival curves and the number of patients. The same investigator assessed the quality of each study using the Newcastle–Ottawa Scale (NOS) [19]. Each study with NOS scores ≥6 was regarded as a high quality study, and studies with NOS scores <6 were regarded as a low quality study.

### Statistical analysis

For this meta-analysis, we measured HR or ORs with 95% confidence for pooled outcomes with a random effects model [20].I^2^ statistic was used to evaluate the heterogeneity of pooled outcomes. If I^2^ >50%, it suggested significant heterogeneity among included studies. To explore other sources of heterogeneity among studies of the OS outcome in this meta-analysis, subgroup analyses of OS according to geographic, research styles and cutoff values were conducted; To validate the credibility of the OS outcome in this meta-analysis, an sensitivity analysis was also performed by removing one study at a time using the ‘‘metaninf” STATA command. Because all pooled outcomes included less than ten trials, publication bias was not evaluated. All statistical analyses were performed using RevMan (version 5.2, Cochrane Library) and Stata 12.0 (StataCorp LP).

## Results

Our search strategy found 85 records. After we removed the duplications by computer and scanned each article by hand, 49 articles were left. We then screened the 49 articles, and 22 studies were identified to be eligible potentially. Ultimately, after reading all 22 studies, 8 full-text articles with a combined 4513 gastric cancer patients were included [5,6,11,13–16,21]. We summarized the characteristics of the included studies in Table 1. All 8 studies were cohort studies (6 retrospective and 2 prospective studies) published between 2010 and 2016.

**Table 1:** Characteristics of included studies in meta-analysis.

First author	Year	Country	Study design	Patients(n)	Age (years)	Treatment	Cut-off value	Study period	NOS score
Aliustaoglu M	2010	Turkey	retrospective	168	60.1±12.1	surgery	>160	2004–2008	6
Deng Q	2015	china	retrospective	389	NA	surgery	≥132	2007–2009	7
Gunaldi M	2015	Turkey	retrospective	245	59.6±11.8	surgery	≥160	NA	7
Jiang N	2014	China	prospective	377	64±11.7	surgery	≥184	2005–2007	8
Kim E Y	2015	Korea	prospective	1986	58.2±11.7	surgery	>126	2000–2009	8
Pang W-y	2016	China	retrospective	392	63(medain)	surgery	>155.67	NA	7
Sun K-y	2015	China	retrospective	632	57(medain)	surgery	> 140	1998–2008	7
Wang D-s	2012	China	retrospective	324	NA	surgery	150/300	2006–2009	7

NA: No available, NOS: Newcastle-Ottawa Scale.

**Fig 2:**
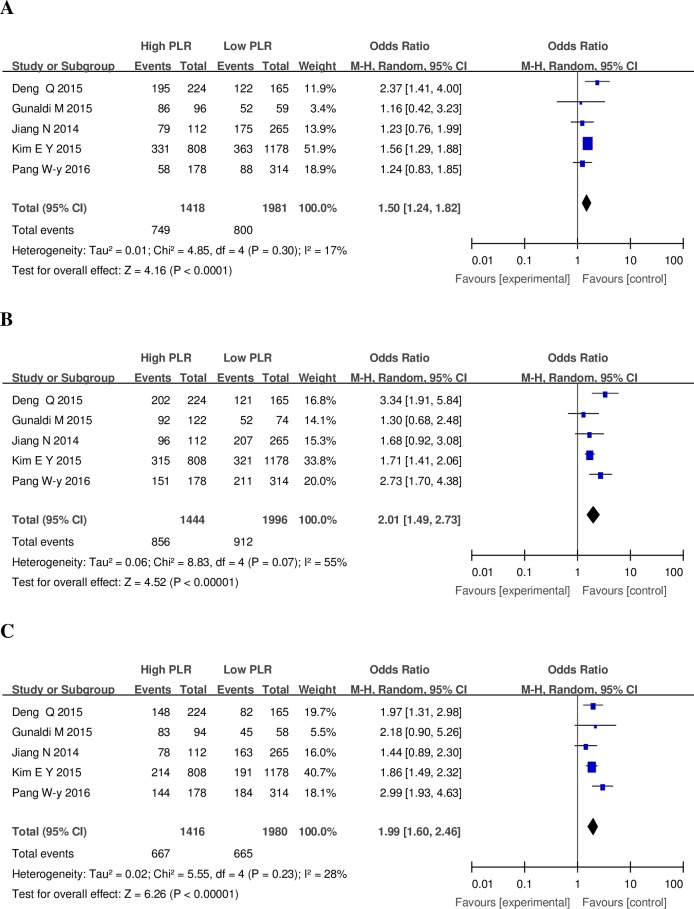
**Forrest plots of included studies evaluating ORs of PLR for lymph node involvement (A), serosal invasion (B), and AJCC staging (C) in gastric cancer.** OR = odds ratio, AJCC = American Joint Committee On Cancer, PLR = platelet-lymphocyte ratio, CI = confidence interval, M-H = Mantel-Haenszel.

**Fig 3:**
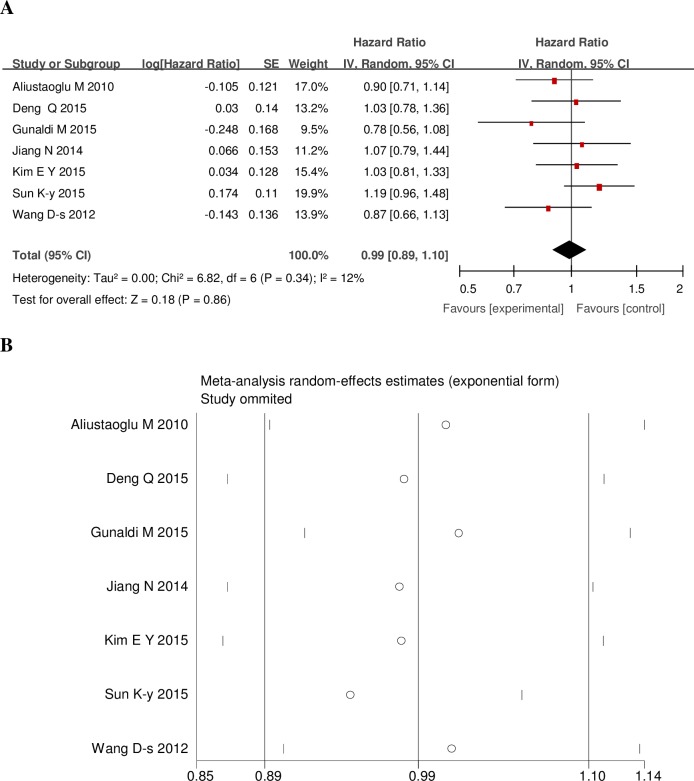
**Forrest plots (A), sensitivity analyses (B) of included studies evaluating HR of PLR for OS.** OS = overall survival, CI = confidence interval, IV = inverse variance, SE = standard error.

In comparison to normal PLR,the high PLR had a higher risk of lymph node metastasis with OR of 1.50 (95% Cl:1.24–1.82; I2 = 17%) (Fig 2A) [6,11,14,15,20]; Coincidentally, the high PLR had a higher risk of serosal invasion (T3 +T4) with OR of 1.99 (95% Cl: 1.60–2.46; I2 = 28%) (Fig 2B) [6,11,14,15,20]; The high PLR also increased the advanced stage (III +IV) risk with OR 1.99 (95% Cl: 1.60–2.46; I2 = 28%) (Fig 2C) [6,11,14,15,20]. An high PLR is not a reliable predictor for OS, however, with an HR of 0.99, it may be a reliable predictor (95%CI: 0.9–1.1; P heterogeneity = 0.34) (Fig 3A) [5,6,11,13–16]. Subgroup analyses of OS according to geographic, research styles and cutoff values were conducted, and these results excluded the potential source of heterogeneity among including studies to some extent (Table 2); we also performed sensitivity analyses for the OS by removing one study at a time to determine whether an individual study influenced the results; there was no significant influence by any single study (Fig 3B). All above results suggested that our results had no obvious heterogeneity between the included studies. All studies with NOS scores ≥6 were regarded as high quality studies.

**Table 2:** Subgroup analyses results.

OS	Variables	No.Trial	No.Patient	Model	HR[95%Cl]	I^2^	Test for subgroup differences I^2^)
	Total	7	4121	Random	0.99 [0.90, 1.10]	12%	No applicable
	Country						31.10%
	China	4	1722	Random	1.05 [0.92, 1.19]	9%	
	Turkey	2	413	Random	0.86 [0.71, 1.04]	0%	
	Korea	1	1986	NA	1.03 [0.81, 1.33]	NA	
	Cut-off						49%
	<160	3	3007	Random	1.10 [0.95, 1.26]	0%	
	≥160	3	790	Random	0.91 [0.78, 1.07]	0%	
	150/300	1	324	NA	0.87 [0.66, 1.13]	NA	
	Research type						0%
	Prospective	2	2363	Random	1.05 [0.86, 1.27]	0%	
	Retrospective	5	1758	Random	0.94 [0.84, 1.06]	37%	

I^2^ statistic was used to evaluate the heterogeneity of pooled outcomes.OS = overall survival, HR = hazard ratio, CI = Confidence interval, NA = No applicable

## Discussion

Many predictive methods have attempted to show an association with the prognosis of various cancers, but these methods may be expensive and unreliable for the prediction of the cancer prognoses. Recently, it was noted that inflammation and the interaction between various inflammatory cells and the extra-cellular matrix plays a crucial role in the tumor micro-environment of tumorigenesis, progression and metastasis [2–4,22,23]. The peripheral blood count can partly reflect the inflammatory response and is routinely conducted with no need for additional effort in patients. In addition, it is convenient and inexpensive [11]. There are two potential interacting mechanisms between inflammation and cancer. First, by generation of reactive oxygen species and proinflammatory cytokines, inflammation may slowly initiate oncogenesis [3,22]. Recently, Qian BZ and Pollard JW demonstrated that at the early stage of the neoplastic progression, inflammation definitely promoted benign neoplasms to cancers [23]. On the other hand, cancer could generate inflammation and that inflammation could then promote low grade malignancies to transition to states of heightened malignancy by genetic evolution [4,24]. Based on these studies, research has attempted to identify the prognostic role of various inflammation-based factors including the PLR, NLR, and the platelet count in cancer patients.

Among various inflammation-based factors, the role of the PLR is the most controversial. Nieswandt B proved that platelets are capable of protecting tumor cells from cytolysis and can promote metastasis. Surface shielding by integrin αIIbβ3 (glycoprotein IIb/IIIa) bridging seems to be the main mechanism of this protection[25], and platelets can also secrete inflammatory proteins such as IL-6, TNF-α, et al, which have also been linked to tumor cell metastases [26,27]. In addition, by the release of secretory factors that promote growth factors, chemokines, proangiogenic regulatory proteins, proteolytic enzymes and microparticles within the microenvironment, activated platelets promote tumor cell growth and invasion [28]. Moreover, Bambace NM demonstrated that platelets might stimulate tumor generation and promote metastasis by creating angiogenic factors, for example platelet-derived growth factor (PDGF) and vascular endothelial growth factor (VEGF) [29]. In addition, a high platelet number would lead to relative lymphocytopenia, and the patient with cancer would have a hypoimmune response that is linked to lymphocyte-mediated antitumor activity at the cellular level.

Based on these above researches, systemic inflammatory parameters, such as the PLR (platelet-lymphocyte ratio), the NLR (neutrophil-lymphocyte ratio) and the platelet count (PLT), have been found to be associated with the prognosis of gastric cancer; however, these results, especially those relating to the PLR, remain inconsistent [5,6,11,13–16]. Although a meta-analysis was conducted and showed that PLR was a negative predictor for gastric cancer in the subgroup, several hundred patients from only 3 articles were included, the 95% confidence intervals were wide, and the conclusion was not realistic with significant heterogeneity [12]. Thus, the prognostic value of the PLR remains inconclusive in gastric cancer. So we aimed to evaluate the prognostic role of the PLR in gastric cancer by conducting and presenting the findings of this meta-analysis. There are two highlights in this analysis. Firstly, blood counts were all derived from the patients’ pretreatment, so it eliminated the influence of the treatment, particularly the chemoradiotherapy influence that can lead to granulocytopenia. Secondly, this is the first meta-analysis about the prognostic role of the PLR in gastric cancer.

This analysis demonstrated that a high PLR is linked to a higher risk of lymph node metastasis, and the high PLR also increased the serosal invasion (T3 +T4) risk and the advanced stage (III +IV) risk in patients with gastric cancer. Although the specific mechanism is still incompletely understood, our results are in accordance with other studies that found that the PLR was a negative prognostic factor for various cancers, such as pancreatic ductal adenocarcinoma, hepatocellular carcinoma and colorectal cancer [30–34]. In this research we found that the PLR could not act as a significant biomarker in the OS of gastric cancer. And subgroup analyses for OS revealed that no differences could distinctly be found in China, Korea or Turkey. Given the various cut off values of the PLR in the included studies, the effects of these different cut off values on the prognostic value of the PLR was evaluated. As well we found that patients with a high PLR did not suffer a worse OS compared to those with a low PLR, regardless of different cut off values. Moreover we also eliminated the effect of different research types on the prognostic value of the PLR. To further improve the pooled result, we used the leave-one-out sensitivity analyses of OS by removing one study at a time to assess if an individual study influenced the results. The result pattern was not obviously impacted by any single study. These results might strengthen the possibility that the PLR cannot be a reliable biomarker in predicting the OS of gastric cancer patients. Some studies also indicated that the PLR is not an independent predictor of cancer OS in gastric cancer. [5,8,35]

There were several limitations in this study. Firstly, this analysis was constrained to only 8 studies and only those published in English, so publication bias could not be excluded. Secondly, there are no randomized controlled trials (RCTs), however, our conclusion is stable with low heterogeneity. Thirdly, many studies in which the local and advanced cancer groups showed an altered PLR after treatment should have been included in our research; however, considering the lack of important clinical parameters, the clinical significance of the PLR could not be further validated. The greatest limitation was the diverse values of the PLR cutoff used in different studies within this meta-analysis. Despite this, the prognostic value of the PLR was not affected, as the majority of the subgroup analysis of the PLR cutoff base on OS did not substantially change the result. Furthermore, a sensitivity analysis was stable from the pooled estimates, which intensified the conclusions. Another limitation is that the HR is heavily dependent on one study [11]. However, if we remove the study, the results remain steady in the analysis. In the future, studies with a larger sample size and more cancer types are needed for more reliable results.

In conclusion, a high PLR was correlated with a higher risk of lymph node metastasis, serosal invasion and advanced stage (III +IV) risk in gastric cancer; However the PLR may not be a significant biomarker in the OS of gastric cancer. We suggest that the PLR could be used before treatment to provide reliable information for patients with gastric cancer.

## Supporting Information

S1 Table Details of literature search in the Databases. (DOCX)

S2 Table PRISMA 2009 Checklist. (DOC)
