# Supplementary material for: The Prognostic Role of the Platelet-Lymphocytes Ratio in Gastric Cancer: A Meta-Analysis
Source: PLoS One. 2016 Sep 29;11(9):e0163719. doi: 10.1371/journal.pone.0163719 (PMC5042439; doi:10.1371/journal.pone.0163719)
Supplement: S1 Table — (DOCX) [file pone.0163719.s001.docx]

**Supporting Information 1: Details of literature search in the Databases**

**Pubmed:**

**(((((“platelet-lymphocyte ratio”[Title/Abstract]) OR “platelet-to-lymphocyte ratio”[Title/Abstract]) OR “platelet lymphocyte ratio”[Title/Abstract]) OR PLR[Title/Abstract])) AND (("Stomach Neoplasms"[Mesh]) OR (((((“gastric cancer”[Title/Abstract]) OR “gastric adenocarcinoma”[Title/Abstract]) OR “gastric carcinoma”[Title/Abstract]) OR "stomach tumor"[Title/Abstract]) OR “stomach neoplasms”[Title/Abstract]))**

**Cochrane Library:**

**#1. "stomach neoplasms":ti,ab,kw or "gastric carcinoma":ti,ab,kw or "gastric adenocarcinoma":ti,ab,kw or "gastric cancer":ti,ab,kw or "stomach tumor":ti,ab,kw (Word variations have been searched) 3443**

**#2. MeSH descriptor: [Stomach Neoplasms] explode all trees 1946**

**#3. #1 or #2 3443**

**#4. "platelet-lymphocyte ratio":ti,ab,kw or "platelet-to-lymphocyte ratio":ti,ab,kw or "platelet lymphocyte ratio":ti,ab,kw or PLR:ti,ab,kw (Word variations have been searched) 49**

**#5. #3 and #4 1**

**Embase:**

**Session Results**

**.......................................................**

**No. Query Results Results Date**

**#12. 'stomach neoplasms':ab,ti OR 'gastric 51 20 Jul 2016**

**carcinoma':ab,ti OR 'gastric**

**adenocarcinoma':ab,ti OR 'gastric cancer':ab,ti**

**OR 'stomach tumor'/exp AND ('platelet-lymphocyte**

**ratio':ab,ti OR 'platelet-to-lymphocyte**

**ratio':ab,ti OR 'platelet lymphocyte ratio':ab,ti**

**OR plr:ab,ti)**

**#11. 'platelet-lymphocyte ratio':ab,ti OR 1,750 20 Jul 2016**

**'platelet-to-lymphocyte ratio':ab,ti OR 'platelet**

**lymphocyte ratio':ab,ti OR plr:ab,ti**

**#10. plr:ab,ti 1,648 20 Jul 2016**

**#9. 'platelet lymphocyte ratio':ab,ti 298 20 Jul 2016**

**#8. 'platelet-to-lymphocyte ratio':ab,ti 347 20 Jul 2016**

**#7. 'platelet-lymphocyte ratio':ab,ti 298 20 Jul 2016**

**#6. 'stomach neoplasms':ab,ti OR 'gastric 125,584 20 Jul 2016**

**carcinoma':ab,ti OR 'gastric**

**adenocarcinoma':ab,ti OR 'gastric cancer':ab,ti**

**OR 'stomach tumor'/exp**

**#5. 'stomach tumor'/exp 125,584 20 Jul 2016**

**#4. 'gastric cancer':ab,ti 59,348 20 Jul 2016**

**#3. 'gastric adenocarcinoma':ab,ti 6,845 20 Jul 2016**

**#2. 'gastric carcinoma':ab,ti 14,189 20 Jul 2016**

**#1. 'stomach neoplasms':ab,ti 161 20 Jul 2016**
